# Gene Expression Analysis with No Sequence Data: Study on Reeves’s Muntjac (*Muntiacus reevesi*)

**DOI:** 10.3390/cimb43030111

**Published:** 2021-10-12

**Authors:** Jadwiga Flaga, Marcin Przybyło, Paweł Górka

**Affiliations:** Department of Animal Nutrition and Biotechnology, and Fisheries, University of Agriculture in Krakow, al. Mickiewicza 24/28, 30-059 Krakow, Poland; m.przybylo@ur.krakow.pl (M.P.); p.gorka@ur.krakow.pl (P.G.)

**Keywords:** primer designing, gene sequence, mRNA expression

## Abstract

Despite scientific progress, the gene sequences for many species not commonly used in research have not yet been analyzed. This makes it difficult to carry out molecular studies on such animals, as the sequence of genes is the basic information used in many techniques. In this study, we attempt to design primers for a real-time PCR analysis, basing on a comparative analysis of selected gene sequences of species related to Reeves’s muntjac (*Muntiacus reevesi*) and by identifying highly conservative regions. Results of PCR products sequencing and their alignment with the GenBank collection show that all selected primers gave products highly similar (> 90%) to the intended target (among compared species), which led us to the conclusion that our primers may be used for further analyses of gene expression.

## 1. Introduction

Real-time PCR is a basic method for a gene expression analysis and is widely used by scientists all over the world [1]. The technique uses DNA polymerase to amplify a specific region of a target nucleic acid and requires specific primers—oligonucleotides that flank the region of interest. Primers are designed on the basis of sequences available in GenBank—a collection of publicly available DNA sequences. The GenBank database is constantly updated; however, its collection is still incomplete. The genomes of relatively few animal species have been fully sequenced and many available sequences, especially for species not commonly used in research, are partial or putative. Thus, the aim of this study is to develop a method of designing primers for a selected gene expression analysis, using real-time PCR, when there is no sequence data available in GenBank for the particular animal. The specific aim of this study is to design and validate primers that will be used for determining the mRNA expression of eleven genes in the tissue samples collected from the gastrointestinal tract (GIT) of Reeves’s muntjac (*Muntiacus reevesi*).

## 2. Materials and Methods

Tissue samples collected from male Reeves’s muntjacs during the realization of a larger research project were used for the purposes of this study (Local Institutional Animal Care and Use Committee, Krakow, Poland, resolution no. 150/2018). In brief, the animals were allocated into a nutritional study and throughout the study period were kept and fed individually one of three experimental diets. The diets consisted of dehydrated chopped lucerne (*ad libitum*), high-fiber pellets (100 g/day) and wheat bran (30 g/day) without (group 0) or with addition of 10 or 20 g of glucose, fructose and sucrose mixture/day (group 10 and group 20, respectively). The GIT samples were collected after 14 days of adaptation to the experimental diets, from four regions of the GIT: atrium ruminis and ventral sac of the rumen (rumen papillae), duodenum and small intestine (whole thickness tissue samples). Tissue samples served for mRNA isolation followed by reverse transcription as described before [2]. The cDNA from each analyzed part of GIT, obtained from four randomly selected animals, was mixed together to create a pooled sample, which served for further analyses.

The mRNA expression of the following genes was of interest: *GAPDH* (glyceraldehyde 3-phosphate dehydrogenase), *ACTB* (beta-actin), *NaKATPase* (sodium–potassium adenosine triphosphatase), *SGLT1* (glucose transporter in rumen and small intestine), *GLUT2* (glucose transporter in small intestine), *GLUT5* (fructose transporter in small intestine), *MCT1* (monocarboxylate transporter 1), *GPR41* and *GPR43* (free fatty acid receptor 3 and 2, respectively), and T1R3 (taste receptor type 1 member 3).

No sequence for aforementioned genes was available for Reeves’s muntjacs in GenBank. Additionally, so far, mRNA expression of aforementioned genes has not been investigated in Reeves’s muntjacs; hence, no literature data were available in this matter. Keeping in mind that gene structure is similar between related species [3], we decided to bring together all sequences for selected genes of species related to Reeves’s muntjacs (suborder *Ruminantia*; infraorder *Pecora*; families *Cervidae* and *Bovidae*; species listed in Supplementary Material, Table S1) which are available in GenBank. For selected genes, we compared all available mRNA sequences (i.e., partial sequences, predicted sequences, transcript variants). Subsequently, we aligned all sequences and identified highly conservative regions (BLAST [4]). For these regions, we designed specific primers using Primer Blast (Table 1).

Primers were designed in such a way that they matched the most conservative sites, avoiding, if possible, even single nucleotide polymorphism (especially within the primer binding sites) and giving the longest possible product (max amplicon length < 250 bp, see Discussion), so that its identification would give the most reliable results. For each gene, at least two primer pairs were designed. These primers served for the real-time PCR analysis where selected genes mRNA expression was determined using StepOnePlus™ Real-Time PCR System (Life Technologies Corporation, Carlsbad, California, USA). Reaction mixture (10 µL of total volume) contained PowerUp™ SYBR^®^ Green Master Mix (Thermo Fisher Scientific, Waltham, MA, USA), 900 nM of each primer (forward or reverse), 1 µL of cDNA sample (0.87 µg) and nuclease-free water (Thermo Fisher Scientific, Waltham, MA, USA). Based on the results of analysis (shape of the amplification curve, purity (based on the melting curve and agarose gel electrophoresis) and quantity (based on the Ct value) of the product) [5], the best primer pair for each gene was chosen. Product of each reaction of the chosen primer pair was sequenced, both forward and reverse. Sequencing was carried out using the Sanger method and BigDye^®^ Terminator v3.1 kit (Life Technologies, Carlsbad, California, USA) in a commercial laboratory (Genomed, Warsaw, Poland). The products of sequencing reactions were separated on a 3730 × 1 DNA Analyzer capillary sequencer (Applied Biosystems, Life Technologies, Carlsbad, California, USA). Chromograms were read in Chromas (version 2.6.6; Technelysium, South Brisbane, Australia). Obtained sequences (Table 2) were used to search GenBank collection for highly similar sequences and, during this alignment, both query coverage and percent identity were considered (Table 3). Subsequently, the efficiency of real-time PCR reaction for each primer pair was checked by creating a relative standard curve using 5-fold serial dilutions of the pooled sample. The total of 4 dilutions was prepared (0.2; 0.04; 0.008; 0.0016) and, together with undiluted sample, they served for making the five-point relative standard curve.

## 3. Results

All selected primers gave products highly similar to the intended targets (Table 3). The query coverage for analyzed PCR products with target gene sequences of compared species was high for the majority of the primers (≥ 90%). Lower values (from 88% to 90%) were observed for both, forward and reverse, products of sequencing for *MCT1* and for the reverse product for *GPR41*. Considering the percent identity, all of the investigated products had a high sequence homology (> 90%) to the intended target, among compared species. The efficiency of reaction for selected primers ranged from 95.13% to 104.46% (mean 100.33% ± 3.00%; Table 1).

## 4. Discussion

A comparative analysis of sequences gives opportunity to decode information contained in DNA of newly sequenced species, to identify coding or noncoding regions and to find regulatory elements [3,6]. However, it usually covers the analysis and processing of already existing sequences. In our study, we tried the opposite approach and attempted to predict how the particular gene sequence might look in the species that have not yet been sequenced.

During the primer designing process, one of the goals was to design primers for the longest possible fragment of coding sequence that was still conservative between species, because the longer the amplicon, the more accurate its identification can be during GenBank collection search. On the other hand, it is generally recommended that the amplicon length for a real-time PCR analysis should be in the range of 50–150 bp or even up to 200 bp [5,7,8,9]. However, research showed that longer amplicons (>200 bp <~250 bp) can still be amplified with a similar efficiency [10,11]. Hence, the maximal length for the designed amplicon in the present study was set at ~250 bp.

Taking into account the above, the longest amplicon we obtained was for *ACTB* (248 bp). As mentioned before, the length of the amplicon may differentially affect the efficiency of the reaction [5,10]. However, in the case of the present study, the efficiency for all analyzed primer sets was very high (in the range of 95–105% [5,12]) and in the case of *ACTB* equaled 103,2%. It was also found that the chemistry used for DNA detection may have an impact on the efficiency of the reaction, with SYBR^®^ Green being less sensitive to the amplicon size than the double-dye probes such as TaqMan^®^ probes [10]. Thus, it is possible that we did not observe loss in reaction efficiency due to the chemistry used.

As shown above, based on the GenBank mRNA sequence database search, all obtained amplicons had a high sequence similarity with the target gene sequences in species related to Reeves’s muntjacs. High sequence identity is considered a good indicator of function similarity [13]. Thus, it led us to the conclusion that the presented method of designing primers seems to be reliable and gives the opportunity to study the expression of genes in less studied animal species.

## Figures and Tables

**Table 1:** Primers used for determination of mRNA expression.

Gene	Gene Description	Forward Primer Reverse Primer	Quantification Cycle (Ct)	Reaction Efficiency	Amplicon Length (bp)
*ACTB*	β-actin	GTCACCAACTGGGACGACAT GTCACCGGAGTCCATCACG	18.84	103.27	246
*GAPDH*	glyceraldehyde-3-phosphate dehydrogenase	TGAGGACCAGGTTGTCTCCT GGCTCAGGGACCTTACTCCT	22.84	100.85	190
*GLUT2*	glucose transporter 2 (*SLC2A2*)	GAGCTATGGCACATCCTGCT TGCTTTGGCTTCCTCATCCA	26.31	97.77	123
*GLUT5*	fructose transporter 5 (*SLC2A5*)	CGTCAGCATTGCCTGTGTCA ACAGTACCCGCCACCATGTA	23.89	100.77	132
*GRP41*	free fatty acid receptor 3 (*FFAR3*)	CTCTTCACCTTCCTCGTGGG CCGAGAGGGTGAGGTTTAGC	27.11	102.56	118
*GRP43*	free fatty acid receptor 2 (*FFAR2*)	TTCTGCTACTCGCGCTTCGT GACCGGCATTGAGGCTACCA	26.85	104.46	214
*MCT1*	solute carrier family 16 (monocarboxylate transporter), member 1 (*SLC16A1*)	TGTTGACATGGTAGCCAGACC AAGGTGCTGCCAGATGACAC	19.92	96.92	125
*NaKATPase*	sodium–potassium adenosine triphosphatase	CCAGGTCTCCGGATTTCACC CACACCCGTGATGATGTGGA	19.72	95.13	218
*SGLT1*	sodium-dependent glucose co-transporter 1 (*SLC5A1*)	TGCTAGGCTGGGTGTTTGTC AGGTAGAGATCCAGGCCCAA	21.64	99.28	205
*T1R3*	taste 1 receptor member 3 (*TAS1R3*)	GAGATCAACAACGCGTCCAC TGCGTGTAGTCACAGTAGGC	27.10	102.34	155

**Table 2:** Results of sequencing of selected primer pairs for the investigated genes.

Gene	Sequencing with Forward Primer	Sequencing with Reverse Primer
*ACTB*	GATAGGCCACCTTCTACACGAGCTGCGTGTGGCCCCCGAGGAGCACCCCGTGCTGCTGACCGAGGCCCCCCTGAACCCTAAGGCCAACCGTGAGAAGATGACCCAGATCATGTTCGAGACCTTCAACACCCCCGCCATGTACGTGGCCATCCAGGCTGTGCTGTCCCTGTATGCCTCTGGCCGCACCACCGGCATCGTGATGGACTCCGGTGACAA	CGAGGCATACAGGGACAGCACAGCCTGGATGGCCACGTACATGGCGGGGGTGTTGAAGGTCTCGAACATGATCTGGGTCATCTTCTCACGGTTGGCCTTAGGGTTCAGGGGGGCCTCGGTCAGCAGCACGGGGTGCTCCTCGGGGGCCACACGCAGCTCGTTGTAGAAGGTGTGGTGCCAGATTTTCTCCATGTCGTCCCAGTTGGTGACA
*GAPDH*	CGACTCACTCTTCTACCTTCGATGCTGGAGCTGGTATTGCCCTCAACGACCACTTTGTCAAGCTCATTTCCTGGTACGACAATGAATTCGGCTACAGCAACAGGGTGGTGGACCTCATGGTCCACATGGCCTCCAAGGAGTAAGGTCCCTGAGCCAAA	TGAGGTCACCACCCTGTTGCTGTAGCCGAATTCATTGTCGTACCAGGAAATGAGCTTGACAAAGTGGTCGTTGAGGGCAATACCAGCTCCAGCATCGAAGGTAGAAGAGTGAGTGTCGCTGTTGAAGTCGCAGGAGACAACCTGGTCCTCAAAA
*GLUT2*	GGCGCATTCTCATGTCTGCTGCTCTTCTTCTGTCCAGAAAGCCCCAGATACCTTTACATCAAGCTGGATGAGGAAGCCAAAGCAA	GTCTGGGCTTTCTGGACAGAAGAGAGCAGCAGACATTGGAGAATGGCTGGCACAGCAGACAAACTAAGCAGGATGTGCCATAGCTCA
*GLUT5*	CATCAGGATGCCTTGGGCCAGTCCCATCCCCGCACTGCTCGTCACTGAGATCTTCCTGCAGTCCTCCCGGCCAGCTGCCTACATGGTGGCGGGTACTGTA	TGCAGGAGATCTCAGTGACGAGCAGTGCGGGGATGGGACTGGGCCCAAGGGCATGCCCTATGACGTAGGAGATGACACAGGCAATGCTGACGA
*GPR41*	CTACTGATGGCCTGGTGATCTTCGTGGGCAAGCTGCGGCGCCGCCCAGTGGCCGTGGATGTGCTCCTGCTAAACCTCACCCTCTCGGA	GATCGGACTGGGCGGCGCCGCAGCTTGCCCACGAAGATCACCAGGGCCATCAGGTTGAGGGGGAGCCCCACGAGGAAGGTGAAGAGA
*GPR43*	CGTGGGGGCTCAGAAGCGGCGCCCAGCCGTGGTGACTGGACATCGTGTCGCTCCTTAATTTCCTGCTGTGCTTTGAGGCCCTATAACATATCCCACCTGGTGGGGTTCCACGTGAAGACAAGCCCCAAGTGGCGGAGGAAAGCTGTGGTGTTTGAGATACGCCTCAATGCCGAATACA	TCTCGCCTTGTGGCTTGCCTTCACGTGGAACCCCACCAGGTGGTATATGTTATAGGGCCCAAAGCACAGCAGGAAATTAAGGAGCGACACTGATGGCCAGTCCCAGGGGTCGTCGCCGCTTCTGAGCCCCCACGTGGGGCTGGGTGAGCATTATCCGCACGAAACGCGAGTAGCAGAAAA
*MCT1*	TTCATGACACACAAAGTGGGTAAGACCTCGAGTTCAGTATTTTTTTGCTGCATCTATTATTGCAAATGGAATGTGTCATCTGGCAGCACCTTAGA	TGATGCAGCAAGAAGTACTGAACTCGAGGTCTTACCCACTTTGTGTTGGCTACAAGTCCCATAGAAGGTGCTGGCTACCGATGTCAACATATAA
*NaKATPase*	ACGAACGGACATTGCCTTTTTTTCACCAACTGTGTTGAAGGCACTGCACGTGGCATTGTTGTGTACACCGGGGATCGAACCGTGATGGGCAGGATTGCGACTCTGGCTTCTGGGCTGGAAGGGGGCCAGACTCCCATCGCTGCAGAAATTGAACATTTTATCCACATCATCACGGGTGTGAA	TTCTGCAGCGATGGGAGTCTGGCCCCCTTCCAGCCCAGAAGCCAGAGTCGCAATCCTGCCCATCACGGTTCGATCCCCGGTGTACACAACAATGCCACGTGCAGTGCCTTCAACACAGTTGGTTGAAAAAAAGGCAATGTTCCGTGTCTCCAGGGGATTTTCATTGGTGAAATCCGGAGACCTGGAGACA
*SGLT1*	CATAGCAGGGTGGTGACGATGCCAGAGTACCTGCGGAAGCGATTTGGAGGCCAGCGGATCCAGGTCTACCTGTCTGTCCTGTCCCTGGTGCTGTACATTTTCACCAAGATCTCGGCAGACATCTTTTCTGGGGCCATATTCATCAACTTGGCCTTGGGCCTGGATCTCTACCTATACC	ATGGCCAGAAGATGTCTGCCGAGATCTTGGTGAAAATGTACAGCACCAGGGACAGGACAGACAGGTAGACCTGGATCCGCTGGCCTCCAAATCGCTTCCGCAGGTACTCTGGCATCGTCACCACCCCTGCTTTAATGTAAATGGGGACAAACACCCAGCCTAGCAATA
*T1R3*	AGCGTCTGGCTATGACCTCTTCGACACATGCTCGGAGCCTGTGGTCGCCATGAAGCCCAGCCTGGTGTTCATGGCCAAAGCGGGCAGCCGCAGCATCGGCGCCTACTGTGACTACACGCAA	CGCTTTGGCCATGACACCAGGCTGGGCTTCATGGCGACCACAGGCTCCGAGCATGTGTCGAAGAGGTCATAGCCCAGACGCAGTCCAGGGAGCAGGGTGGACGCGTTGTTGATCTCA

**Table 3:** Result of alignment of sequenced PCR reaction products with GenBank mRNA sequence database.

Gene	Product Length/Primer		Item ^1^	*Odocoileus Virginianus Texanus*	*Bos Taurus*	*Bos Mutus*	*Bos Indicus*	*Bison Bison*	*Bubalus Bubalis*	*Syncerus Caffer*	*Ovis Aries*	*Capra Hircus*	Mean
*ACTB*	246 bp ^2^	F	q cover	95.00	95.00	95.00	95.00	95.00	95.00	95.00	95.00	95.00	95.00
			per ident	99.52	97.60	97.60	96.63	97.12	95.19	96.15	96.63	97.60	97.12
		R	q cover	99.00	99.00	99.00	99.00	99.00	-	99.00	99.00	99.00	99.00
			per ident	99.52	97.61	97.61	96.65	97.61	-	96.65	97.14	97.61	97.55
*GAPDH*	190 bp	F	q cover	96.00	94.00	94.00	94.00	94.00	94.00	94.00	92.00	94.00	94.00
			per ident	98.69	97.99	98.66	97.99	98.66	98.66	98.66	98.63	97.99	98.44
		R	q cover	98.00	98.00	98.00	98.00	98.00	98.00	98.00	98.00	98.00	98.00
			per ident	98.03	97.37	98.03	97.37	98.03	98.03	98.03	98.03	97.37	97.81
*GLUT2*	123 bp	F	q cover	95.00	95.00	100.00	95.00	100.00	95.00	-	95.00	95.00	96.25
			per ident	97.59	96.39	95.40	96.39	95.40	96.39	-	96.39	96.39	96.29
		R	q cover	97.00	97.00	97.00	97.00	97.00	97.00	-	97.00	98.00	97.13
			per ident	96.55	94.25	93.10	94.25	93.10	95.40	-	96.55	96.59	94.97
*GLUT5*	132 bp	F	q cover	92.00	92.00	92.00	92.00	92.00	92.00	-	92.00	92.00	92.00
			per ident	97.87	95.74	96.81	95.74	96.81	96.81	-	97.87	97.87	96.94
		R	q cover	98.00	98.00	98.00	96.00	98.00	98.00	-	98.00	97.00	97.63
			per ident	98.92	95.70	96.77	95.60	96.77	96.77	-	97.85	96.74	96.89
*GPR41*	118 bp	F	q cover	96.00	96.00	96.00	96.00	96.00	96.00	-	96.00	96.00	96.00
			per ident	96.51	93.02	94.19	93.02	94.19	94.19	-	95.35	94.19	94.33
		R	q cover	88.00	88.00	88.00	88.00	88.00	88.00	-	88.00	88.00	88.00
			per ident	100.00	100.00	100.00	100.00	98.70	100.00	-	100.00	100.00	99.84
*GPR43*	214 bp	F	q cover	96.00	94.00	94.00	94.00	94.00	94.00	-	94.00	94.00	94.25
			per ident	93.60	89.94	90.53	89.94	89.94	89.94	-	90.53	89.94	90.55
		R	q cover	95.00	95.00	95.00	95.00	95.00	95.00	-	96.00	96.00	95.25
			per ident	93.60	90.12	90.70	90.12	90.12	89.53	-	89.60	89.02	90.35
*MCT1*	125 bp	F	q cover	88.00	88.00	88.00	88.00	88.00	88.00	-	88.00	88.00	88.00
			per ident	100.00	96.43	96.43	96.43	95.24	96.43	-	97.62	96.43	96.88
		R	q cover	93.00	78.00	93.00	78.00	93.00	93.00	-	93.00	93.00	89.25
			per ident	94.38	97.30	92.13	97.30	92.13	92.13	-	93.26	93.26	93.99
*NaKATPase*	218 bp	F	q cover	96.00	96.00	96.00	96.00	96.00	96.00	-	96.00	96.00	96.00
			per ident	97.75	93.82	93.82	93.26	93.82	93.26	-	96.63	97.19	94.94
		R	q cover	97.00	97.00	97.00	97.00	97.00	97.00	-	97.00	97.00	97.00
			per ident	98.92	95.14	95.14	94.59	95.14	94.05	-	96.76	97.30	95.88
*SGLT1*	205 bp	F	q cover	95.00	93.00	93.00	93.00	93.00	93.00	-	91.00	91.00	92.75
			per ident	99.42	100.00	99.40	100.00	99.40	99.40	-	99.39	99.39	99.55
		R	q cover	93.00	93.00	93.00	93.00	93.00	93.00	-	94.00	93.00	93.13
			per ident	99.36	98.09	97.45	98.09	97.45	97.45	-	97.47	97.45	97.85
*T1R3*	155 bp	F	q cover	99.00	98.00	98.00	98.00	98.00	95.00	-	98.00	98.00	97.75
			per ident	97.52	93.33	94.17	93.33	94.17	94.02	-	93.33	94.17	94.26
		R	q cover	99.00	98.00	98.00	98.00	98.00	98.00	-	95.00	95.00	97.38
			per ident	96.58	91.38	93.10	92.24	93.10	93.10	-	94.69	94.69	93.61

^1^ q cover—query coverage (%); per ident—percent identity; ^2^ bp—base pairs.

## Data Availability

Data is contained within the article and Supplementary Material.

## Supplementary Materials

The following are available online at https://www.mdpi.com/article/10.3390/cimb43030111/s1, Table S1: Contains the list of sequences (with GenBank accession numbers) used for the comparative analysis of selected genes.
