# Supplementary material for: Gene Expression Analysis with No Sequence Data: Study on Reeves’s Muntjac (Muntiacus reevesi)
Source: Curr Issues Mol Biol. 2021 Oct 12;43(3):1576–82. doi: 10.3390/cimb43030111 (PMC8929141; doi:10.3390/cimb43030111)
Supplement: Supplementary file 1 [file cimb-43-00111-s001.zip › cimb-1404316-supplementary.pdf]

Suppl. Table S1. contains the list of sequences (with GenBank accession numbers) used for the comparative analysis of selected genes.

Table S1. Sequences of selected genes used for the comparative analysis

Gene	Gene description	Organism	GenBank Accesion no.	Type of sequence
ACTB	beta-actin	*Odocoileus virginianus*	XM_020874713.1	predicted mRNA
		Bos taurus	NM_173979.3	mRNA
		Capra hircus	NM_001314342.1	mRNA
		Ovis aries	NM_001009784.3	mRNA
		Cervus elaphus	DQ233465.1	partial cds
GAPDH	glyceraldehyde-3-phosphate dehydrogenase	*Odocoileus virginianus*	XM_020878738.1	predicted mRNA
		Bos taurus	NM_001034034.2	mRNA
		Capra hircus	XM_005680968.3	predicted mRNA
		Ovis aries	NM_001190390.1	mRNA
		Cervus elaphus	AY650282.1	partial cds
GLUT2	glucose transporter 2	*Odocoileus virginianus*	XM_020898839.1	predicted transcript 1
			XM_020898840.1	predicted transcript 2
		Bos taurus	NM_001103222.1	mRNA
			XM_005201668.4	predicted transcript 1
		Capra hircus	XM_005675321.3	predicted mRNA
		Ovis aries	XM_004003162.4	predicted mRNA
GLUT5	fructose transporter 5	*Odocoileus virginianus*	XM_020876860.1	predicted transcript 1
			XM_020877582.1	predicted transcript 2
		Bos taurus	NM_001101042.2	mRNA
			XM_025001418.1	predicted transcript 1
		Capra hircus	MH308027.1	partial cds
		Ovis aries	NM_001009451.1	mRNA
			XM_027975571.1	predicted transcript 1
GPR41	free fatty acid receptor 3	*Odocoileus virginianus*	XM_020871822.1	predicted mRNA
		Bos taurus	NM_001145233.1	mRNA
			XM_015458060.2	predicted transcript 1
		Capra hircus	NM_001285653.1	mRNA
			XM_005692314.3	predicted transcript 1
		Ovis aries	XM_027977432.1	predicted mRNA
GPR43	free fatty acid receptor 2	*Odocoileus virginianus*	XM_020871694.1	predicted mRNA
			XM_020871823.1	predicted transcript 1
			XM_020871824.1	predicted transcript 2
		Bos taurus	NM_001163784.1	mRNA
			XM_005218963.4	predicted transcript 1
			FJ562212.1	mRNA
		Capra hircus	NM_001285655.1	mRNA
			HM623658.1	mRNA
		Ovis aries	XM_012190178.3	predicted mRNA
		Bison bison	XM_010849004.1	predicted mRNA
		Bubalus bubalis	XM_006068814.2	predicted transcript 1
			XM_025269947.1	predicted transcript 2
MCT1		*Odocoileus virginianus*	XM_020871478.1	predicted transcript 1
			XM_020871479.1	predicted transcript 2
			XM_020871480.1	predicted transcript 3
			XM_020871481.1	predicted transcript 4
			XM_020871482.1	predicted transcript 5
			XM_020871483.1	predicted transcript 6
		Ovis aries	XM_004002335.3	predicted transcript 1
			XM_015092176.1	predicted transcript 2
			XM_012178807.2	predicted transcript 3

			XM_012178811.2	predicted transcript 4
			XM_012178817.2	predicted transcript 5
		Bos taurus	NM_001037319.1	mRNA
			XM_024986151.1	predicted transcript 1
			XM_015463657.2	predicted transcript 2
NaKATPase	sodium–potassium adenosine triphosphatase	*Odocoileus virginianus*	XM_020892330.1	predicted mRNA
		Bos taurus	NM_001076798.1	mRNA
		Capra hircus	XM_018045956.1	predicted mRNA
		Ovis aries	NM_001009360.1	mRNA
			XM_027968332.1	predicted transcript 1
SGLT1	sodium-dependent glucose co-transporter 1	*Odocoileus virginianus*	XM_020871612.1	predicted transcript 1
			XR_002309549.1	predicted transcript 2
			XM_020871613.1	predicted transcript 3
			XM_020871614.1	predicted transcript 4
			XM_020871615.1	predicted transcript 5
		Bos taurus	NM_174606.2	mRNA
		Capra hircus	NM_001314323.1	mRNA
			XM_018060890.1	predicted transcript 1
		Ovis aries	NM_001009404.1	mRNA
T1R3	taste 1 receptor member 3	*Odocoileus virginianus*	XM_020873827.1	predicted mRNA
		Bos taurus	XM_024976809.1	predicted transcript 1
			XM_024976810.1	predicted transcript 2
		Capra hircus	XM_018060532.1	predicted mRNA
		Ovis aries	XM_027975813.1	predicted mRNA
		Bison bison	XM_010849004.1	predicted mRNA
		Bubalus bubalis	XM_006068814.2	predicted transcript 1
			XM_025269947.1	predicted transcript 2
